# A case report of thyroid sarcomatoid carcinoma

**DOI:** 10.1097/MD.0000000000051191

**Published:** 2026-10-23

**Authors:** Lei Zhang, Lijing Ran, Lulu Zhao, Linxue Qian

**Affiliations:** a Department of Ultrasound, Beijing Friendship Hospital, Capital Medical University, Beijing, China; b Department of Ultrasound, Affiliated Hospital of Jining Medical University, Jining, China; c Clinical Medical College, Jining Medical University, Jining, China.

**Keywords:** thyroid sarcomatoid carcinoma, ultrasound

## Abstract

**Rationale:**

Sarcomatoid carcinoma represents a rare subtype of highly aggressive malignant neoplasm. Sarcomatoid carcinoma of the thyroid is extremely uncommon, accounting for merely 1% to 2% of all thyroid malignancies. It is marked by rapid progression and unfavorable prognosis, with a median survival of only 5 to 6 months. Additional case data are required to assist clinicians in the diagnosis and treatment of this rare disorder.

**Patient concerns:**

A 65-year-old male presented with fever and left cervical pain. Physical examination identified a hard palpable mass within the left thyroid lobe.

**Diagnoses:**

Thyroid ultrasonography revealed a hypoechoic nodule within the left lobe, with an anterior-posterior-to-transverse diameter ratio >1; circumferential calcification and scattered intranodular blood flow signals were observed. Additionally, enlarged and fused left cervical lymph nodes were identified. Computed tomography demonstrated that a left thyroid mass was compressing the trachea. A percutaneous fine-needle aspiration biopsy combined with immunohistochemical staining (showing positivity for both cytokeratin and vimentin) ultimately confirmed the diagnosis of thyroid sarcomatoid carcinoma.

**Interventions:**

Following multidisciplinary team consultation, the patient underwent 4 cycles of combination therapy with sintilimab, paclitaxel, and anlotinib.

**Outcomes:**

The patient achieved disease remission after combination therapy but ultimately died of disease progression 9 months post-diagnosis.

**Lessons:**

This sarcomatoid carcinoma of the thyroid case presents typical clinical and ultrasonographic manifestations. Pathological assessment combined with immunohistochemistry facilitates definitive diagnosis. The adopted combination regimen yielded temporary disease control. This case provides valuable references for the individualized diagnosis and management of this rare thyroid malignancy.

## 1. Introduction

Sarcomatoid carcinoma is a rare and highly aggressive malignant neoplasm,^[1]^ also known as spindle cell carcinoma, pseudosarcoma, and carcinosarcoma. Its pathogenesis remains poorly elucidated, and this tumor predominantly arises in the lungs, esophagus, ovaries, and other organs.^[2]^ Sarcomatoid carcinoma of the thyroid (STC) is even rarer. Classified as a subtype of anaplastic thyroid carcinoma (ATC), STC exhibits an extremely low clinical incidence, accounting for merely 1% to 2% of all thyroid malignancies, while thyroid cancers constitute approximately 3.6% of all human neoplasms.^[3]^ This disease mainly affects elderly individuals with a predominant onset age ranging from 60 to 70 years, and it is more prevalent in females with a male-to-female ratio of approximately 1:5.^[4]^ STC possesses extremely high malignant potential and an exceedingly poor clinical prognosis accompanied by a high mortality rate, with a median survival time of only 5 to 6 months. Merely 10% to 15% of patients can achieve a survival duration exceeding 2 years after comprehensive antitumor therapy.^[3]^

### 1.1. Ethical approval

This study has received ethical approval from the Medical Scientific Research Ethics Committee of the Affiliated Hospital of Jining Medical University (Approval No.: 2025-10-C005). The conduct of this study strictly adheres to the principles of the Declaration of Helsinki.

## 2. Case presentation

A 65-year-old male patient presented with fever combined with left cervical pain and was initially diagnosed with subacute thyroiditis. Specialized physical examination revealed a full neck. A mass, hard in texture and slightly tender to pressure, was palpable on the left lobe of the thyroid gland during swallowing, which is hard in texture and slightly tender to pressure. Multiple enlarged lymph nodes were palpable in the left cervical regions III and IV. Laboratory tests showed a C-reactive protein of 15.94 mg/L, an erythrocyte sedimentation rate of 19.00 mm/h, and a white blood cell count of 12.31 × 10^9^ L^−1^. Three thyroid function tests indicated free triiodothyronine of 6.22 pmol/L, free thyroxine of 21.70 pmol/L, thyroid-stimulating hormone of 3.27 mIU/L, thyrotropin receptor antibody of 1.00 IU/L, and thyroglobulin antibody of 15.800 IU/mL. Carcinoembryonic antigen was 2.52 ng/mL.

Thyroid ultrasound: A solid hypoechoic nodule in the left lobe of the thyroid gland, approximately 3.5 cm × 4.4 cm × 2.6 cm (left-right diameter × anterior-posterior diameter × superior-inferior diameter), with a longitudinal-to-transverse ratio >1. A ring-shaped calcification was seen inside (Fig. 1A), and sparse blood flow signals were noted (Adler grade 1), RI:0.67 (Fig. 1B). Multiple enlarged lymph nodes were observed in the left neck. These lymph nodes were mutually fused, and the hilar structure of the lymph nodes had disappeared (Fig. 1C). Ultrasound findings indicated a solid hypoechoic nodule in the left lobe of the thyroid gland, classified as C-TIRADS category 4C (refer to the《2020 Chinese Guidelines for Ultrasound Malignancy Risk Stratification of Thyroid Nodules: C-TIRADS》. Endocrine 70, 256–279 (2020).) . Additionally, multiple enlarged lymph nodes are present in the left neck, with a potential for metastasis. Neck computed tomography (CT) scan reveals a soft tissue density shadow in the left lobe of the thyroid gland, with the trachea being compressed. Enlarged and fused lymph nodes were observed on the left side of the neck (Fig. 1D). Thyroid static imaging indicated no obvious radionuclide accumulation in the left lobe of the thyroid gland (Fig. 1E). A histological biopsy was performed on the nodule in the left lobe of the thyroid gland, and the pathological diagnosis was thyroid sarcomatoid carcinoma. Microscopic findings showed a malignant tumor with necrosis, mostly exhibiting high-grade sarcomatous changes (Fig. 1F). Immunohistochemistry results were cytokeratin (CK) (+), Vimentin (+), TG (−), CK7 (−), CD31 (−).

**Figure 1.**
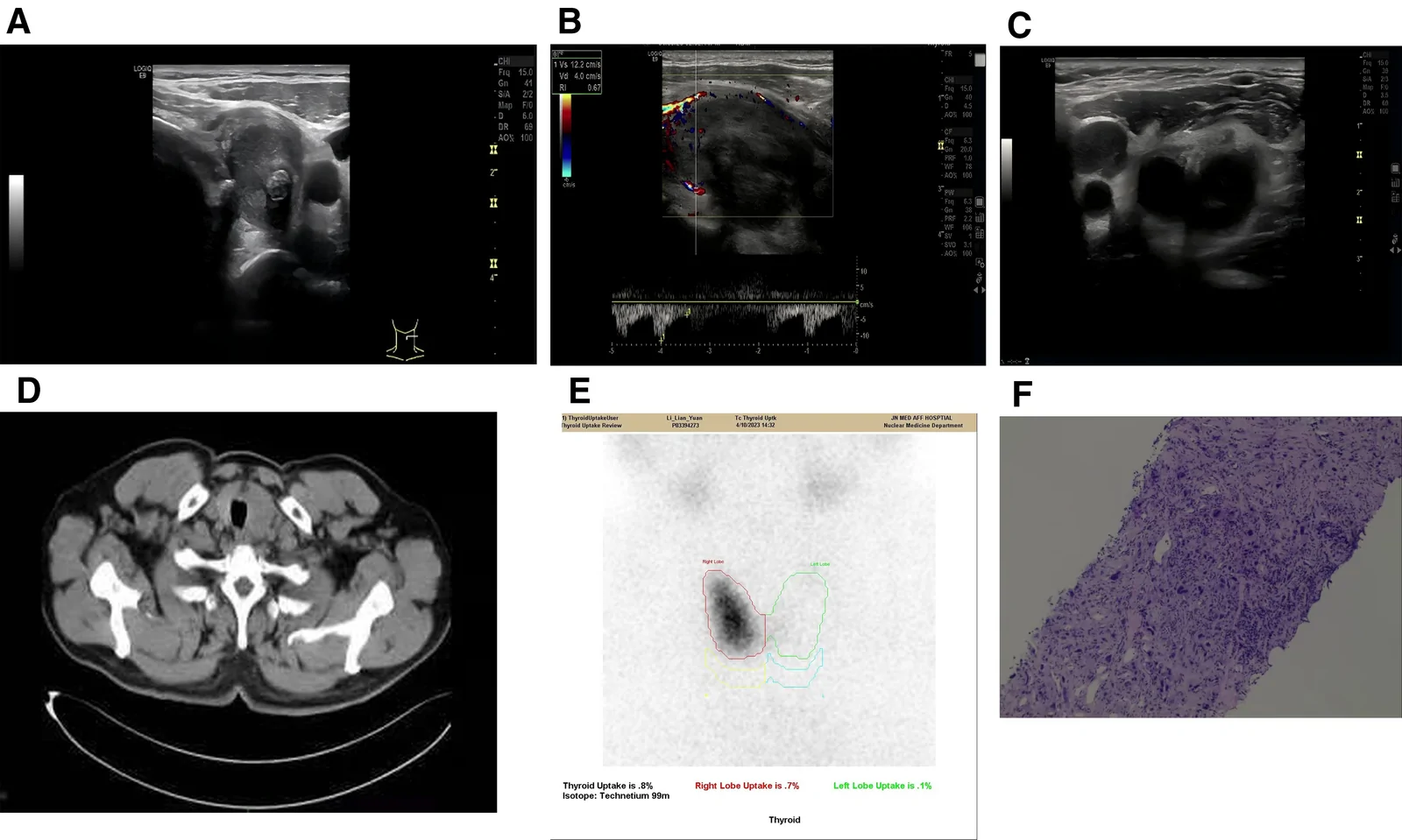
A (A solid hypoechoic nodule in the left lobe of the thyroid gland with an aspect ratio >1, containing circumferential calcifications). B (Scattered blood flow signals were observed within the nodule (Adler grade 1); RI: 0.67). C (Multiple enlarged lymph nodes are observed in the left cervical region, which are fused together, with the lymphatic portal structure absent). D (CT of the neck reveals a soft tissue density shadow in the left lobe of the thyroid gland, tracheal compression, and enlarged lymph nodes with fusion in the left cervical region). E (Static thyroid imaging revealed no significant radionuclide uptake in the left lobe of the thyroid gland). F (Microscopic findings: Malignant tumors with necrosis, most exhibiting high-grade sarcomatous changes. HE, ×100). CT = computed tomography.

Following multidisciplinary team discussion, the patient received 4 cycles of combined therapy comprising sintilimab-based immunotherapy, chemotherapy, and targeted therapy. Subsequent reevaluation via thyroid ultrasonography (Fig. 2A) and cervical CT (Fig. 2B) revealed remarkable tumor regression. The patient died of disease progression at the 9-month follow-up after combined therapy.

**Figure 2.**
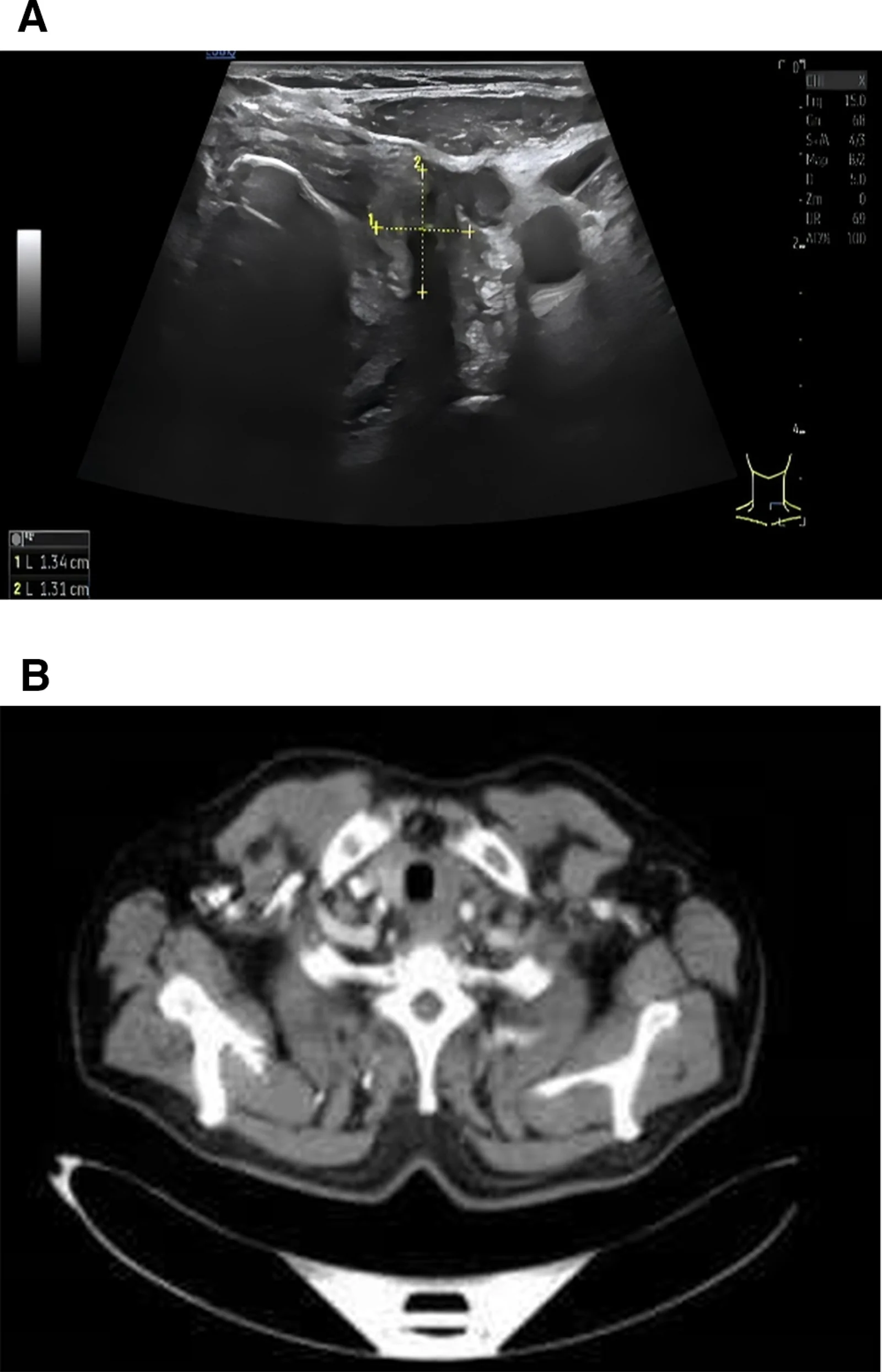
A (Follow-up thyroid ultrasound after combined therapy showed significant reduction in tumor size). B (Follow-up neck CT after combined therapy showed significant tumor reduction and improved tracheal compression). CT = computed tomography.

### 2.1. Written informed consent statement

The patient in this case report is deceased. All reasonable attempts were made to contact the patient’s next of kin for publication consent. No identifiable personal information is presented in the manuscript, and all data have been thoroughly anonymized to protect privacy. There is no foreseeable risk of personal identification or ethical conflict with publication.

## 3. Discussion and conclusions

STC is classified as a subtype of ATC, and its exact etiology remains incompletely clarified. This neoplasm is more prevalent in iodine-deficient regions and in individuals with a prior history of thyroid malignancy. Multiple postoperative recurrences of thyroid tumors and a history of cervical radiation exposure are regarded as potential predisposing factors.^[5]^ Studies have verified that STC is a biphasic neoplasm of monoclonal origin.^[6]^ It is presumably derived from thyroid follicular epithelial cells and mostly develops from differentiated thyroid carcinoma via dedifferentiation associated with sarcomatous transformation. Genetic investigations^[7–9]^ have demonstrated that mutations in tumor suppressor genes and oncogenes, including p53, β-catenin, NRas, and *PTEN*, as well as specific gains and losses of certain chromosomal regions such as 3p13–14+, 5q11–31–, 11q13+, 8p, and 8q, exert pivotal effects on this neoplastic transformation process. Although several cases present purely sarcomatoid features at initial diagnosis, STC shares conserved core genetic mutations with differentiated thyroid carcinomas, which supports the multistep tumor progression and dedifferentiation-driven pathogenesis of these 2 malignancies.^[10]^ Furthermore, epithelial-mesenchymal transition (EMT) also plays a crucial role in the pathogenesis of STC. Epithelial-mesenchymal transition is defined as a biological process wherein epithelial cells acquire mesenchymal phenotypes under sequential programmed regulatory mechanisms,^[11]^which has been well documented to be tightly correlated with tumor metastasis.^[12]^

Clinically, STC is mainly characterized by rapidly enlarging cervical masses that develop over a short period. Most lesions present as solitary nodules, which are typically hard in texture, irregular in surface, ill-defined in boundary, and poor in mobility, features consistent with the typical manifestations of malignant nodules. STC progresses rapidly and is challenging to diagnose at an early stage. Approximately 15% to 50% of patients present with tumor extension beyond the thyroid gland at the time of initial diagnosis.^[5]^ The common clinical symptoms are caused by local tumor compression or invasion of surrounding tissues: invasion of the trachea leads to dyspnea or hemoptysis, invasion of the esophagus results in dysphagia, and invasion of the recurrent laryngeal nerve induces hoarseness^[13]^; approximately 25% of cases are characterized by bilateral thyroid involvement. A small proportion of patients initially present with distant metastases. The lungs and pleura are the most common metastatic sites, while the adrenal glands, gastrointestinal tract, bones, and brain may also be involved. In this case, an enlarged mass was identified in the left lobe of the thyroid gland, which was hard in texture and accompanied by mild tenderness. The mass caused tracheal compression and was associated with ipsilateral cervical lymph node metastasis, with no evidence of distant metastasis. Laboratory tests revealed elevated white blood cell count and erythrocyte sedimentation rate in the patient. The preliminary diagnosis included upper respiratory tract infection, which responded well to anti-infective therapy. Therefore, the fever was attributed to upper respiratory tract infection rather than tumor necrosis.

STC lesions are generally large in size, with fish-flesh-like cut surfaces ranging from whitish to tan in color, and frequently accompanied by hemorrhage and necrosis. Pathologically, this tumor is classified into 2 histological subtypes: The tumor retains epithelial morphological characteristics without the formation of follicular, papillary, or nested architectures and it predominantly presents with sarcomatoid histological architecture. Histologically, STC exhibits extensively invasive growth patterns and is composed of diverse cellular components, including spindle cells, pleomorphic giant cells, and epithelioid cells, among which spindle cells constitute the predominant population. The present case was confirmed as the sarcomatoid subtype, which displayed highly malignant morphological features, including pronounced nuclear pleomorphism and abundant mitotic figures. Distinct stromal necrosis and inflammatory infiltration were also observed. The lesion was predominantly composed of malignant spindle cells, exhibiting histological characteristics analogous to pleomorphic sarcoma.^[14]^

Given the rarity of ATC and that most lesions tend to attain a large size, CT serves as the predominant imaging examination for its clinical evaluation.^[15]^ Currently, literature focusing on ultrasonic imaging features remains extremely scarce, and relevant imaging studies concerning STC are barely reported. In the present case, STC manifested as a hypoechoic nodule with heterogeneous internal echo and marginal circular calcification on ultrasonography, which was consistent with the sonographic features of ATC reported by Gu et al^[16]^ Hahn et al^[17]^ retrospectively analyzed ultrasonographic findings of 26 patients diagnosed with ATC and recorded a mean tumor diameter of 4.2 cm. Their study concluded that ATC typically presents with irregular shapes and ill-defined borders on ultrasound, which further complements the imaging manifestations observed in the present case. Combined with previous literature and clinical experience, the ultrasonographic characteristics of STC are summarized as follows: solid hypoechoic nodules with vertical orientation, heterogeneous internal echoes accompanied by calcification (rim calcification was detected in this case), poor blood flow perfusion, and regional lymphadenopathy in view of its rapid disease progression.^[18]^ Ultrasonography and CT offer certain reference values for the diagnosis of STC, yet they cannot serve as definitive diagnostic criteria. Undifferentiated cancer lesions are large, often involving an entire lobe, with rich blood flow signals. Some lesions have clear boundaries and regular shapes, appearing as cystic-solid. Papillary carcinoma typically shows on an ultrasound as a solid, low-echo nodule with tiny, sand-like calcifications. It has an irregular shape and unclear borders, with relatively rich blood flow inside the lesion and is accompanied by enlarged lymph nodes in the neck. Medullary carcinoma usually appears on ultrasound as a single, mainly solid hypoechoic nodule, with an irregular shape and clear boundaries, no halo around it, and small or large calcifications inside, showing the typical ultrasound features of thyroid cancer. The follicular carcinoma nodule shows low echogenicity, unclear borders, irregular shape, no halo, tiny calcifications, and abundant blood flow signals inside the nodule.

Final confirmation relies on pathological manifestations and immunohistochemical examinations. Specifically, CK and mesenchymal marker vimentin are of pivotal significance in establishing its diagnosis. Vimentin is predominantly localized in the cytoplasm, which modulates cell migration and invasion and participates in cellular signal transduction. It is closely correlated with tumor invasion, metastasis, and unfavorable clinical prognosis.^[19]^ In the immunohistochemical detection of STC, pan-CK is the most prevalent epithelial biomarker with a positive expression rate ranging from 40% to 100%. Positive expression of thyroid transcription factor–1 indicates the thyroid origin of tumors, while negative thyroglobulin expression facilitates differential diagnosis from differentiated thyroid carcinomas. Conversely, sporadic thyroglobulin positivity is generally ascribed to the entrapment of nonneoplastic thyroid tissues or residual well-differentiated tumor components. ^[20]^ Immunohistochemical results of the present case revealed positive expression of CK and vimentin, suggestive of anaplastic carcinoma, which was consistent with previous literature findings. Nevertheless, the positive rate of CK in STC is merely 26% ^[2]^ and comprehensive evaluation of multiple biomarkers is essential to avoid missed diagnosis.

Surgical resection serves as a principal therapeutic modality for STC. For early-stage patients without extrathyroidal invasion and lymph node metastasis, bilateral total thyroidectomy combined with lymphadenectomy is recommended, followed by adjuvant chemotherapy after surgery. Several patients are expected to achieve a survival duration exceeding 5 years.^[21]^ In cases with peritumoral tissue invasion, regional debulking resection is advisable, followed by postoperative combined radiotherapy and chemotherapy to prolong patient survival.^[3]^ For patients ineligible for surgical intervention, radiotherapy alone yields the poorest therapeutic efficacy regardless of metastatic status, and single-agent chemotherapy or concurrent chemoradiotherapy is thus strongly recommended.^[3]^ At present, there is still a lack of unified standardized chemotherapy regimens for this disease. Chemotherapy is primarily adopted as bridging therapy or a definitive therapeutic strategy, and the mainstream chemotherapeutic agents encompass doxorubicin, cisplatin, docetaxel, and paclitaxel.^[22]^ In accordance with the 2021 American Thyroid Association *Guidelines for the Management of Patients with ATC*, targeted therapy and immunotherapy have gradually emerged as mainstream therapeutic approaches for advanced ATC.^[23]^ Accumulating evidence has validated that genetic aberrations involving *BRAF*, *RAS*, *PI3K*, *PTEN*, and Notch are tightly implicated in the oncogenesis and progression of ATC, which provide promising molecular targets for targeted therapeutic strategies.^[24,25]^ Hui et al ^[10]^ reported 1 case achieving stable disease status following antitumor therapy with anlotinib, a multi-targeted tyrosine kinase inhibitor. Another patient with extensive distant metastasis sequentially received combined regimens consisting of recombinant human endostatin plus docetaxel-capecitabine, as well as nimotuzumab combined with cyclophosphamide-doxorubicin-cisplatin. Tumor lesions were markedly shrunk after concomitant nutritional supportive care. Although comprehensive interventions including thyroidectomy combined with radiotherapy, chemotherapy, targeted therapy, and immunotherapy can optimize clinical outcomes, the overall 5-year survival rate remains below 10%. Al-Qurayshi et al ^[26]^retrospectively analyzed over 210,000 adult patients diagnosed with thyroid carcinoma enrolled in *the National Cancer Database* from 2004 to 2014. Among these cases, 1870 were confirmed as ATC, accounting for approximately 0.9% of the total cohort. The 2-year survival rate reached 27.6% in patients without tumor metastasis, whereas it dropped to merely 8.01% in those presenting with metastatic lesions. This case exhibited high PD-L1 expression; no *BRAF* gene mutations were detected; and after consultation with specialists from a higher-level hospital, triple therapy (sintilimab + paclitaxel + anlotinib) was administered.After receiving 4 cycles of combined therapy, the patient exhibited a significant reduction in tumor lesions, demonstrating the efficacy of this novel treatment regimen. The 9-month survival period exceeded the previously reported range of 5 to 6 months. However, due to confirmed cervical lymph node metastasis indicating advanced-stage disease, the patient ultimately died from multi-organ metastasis.

Furthermore, reviewing the diagnostic timeline of this case: On April 10, 2023, the patient was initially diagnosed with subacute thyroiditis due to concurrent fever symptoms and hospitalized for treatment. During hospitalization, ultrasound findings suggested a suspected malignant thyroid lesion; however, the patient declined a thyroid biopsy. At a follow-up visit on May 5, 2023, the patient agreed to undergo a thyroid biopsy, and a diagnosis of thyroid ATC was confirmed on May 8, 2023. The period from initial presentation to definitive diagnosis lasted nearly 1 month. The diagnostic delay resulted from both an initial misdiagnosis and the patient’s refusal to undergo biopsy, representing a clinical lesson worthy of reflection.

As a rare and highly aggressive thyroid malignancy, STC remains incompletely elucidated in its pathogenic mechanisms. It is characterized by difficult early diagnosis, limited therapeutic options, and extremely poor clinical prognosis. Accordingly, the overall 5-year survival rate remains below 10%. In-depth exploration of its pathogenesis and pathological features, improvement of early diagnostic efficacy, and development of novel therapeutic strategies are of great clinical significance for optimizing patient prognosis. Meanwhile, accumulating sufficient clinical cases and summarizing relevant diagnostic and therapeutic experiences can lay a solid foundation for establishing individualized precise management regimens.

## Author contributions

**Investigation:** Lijing Ran.

**Methodology:** Lulu Zhao, Linxue Qian.

**Resources:** Lulu Zhao.

**Writing – review & editing:** Linxue Qian.

**Writing – original draft:** Lei Zhang.
